# Investigating the role of Epstein-Barr virus and human papillomavirus types 16 and 18 co-infections in cervical disease of Iranian women

**DOI:** 10.3389/fonc.2024.1331862

**Published:** 2024-04-24

**Authors:** Farzane Sadeghi, Talieh Mostaghimi, Mahdie Taheri, Shahla Yazdani, Maryam Javadian, Mohammad Ranaee, Hossein Ghorbani, Zinatossadat Bouzari, Farzin Sadeghi

**Affiliations:** ^1^ Student Research Committee, Babol University of Medical Sciences, Babol, Iran; ^2^ Department of Microbiology, School of Medicine, Golestan University of Medical Sciences, Gorgan, Iran; ^3^ Cancer Research Center, Health Research Institute, Babol University of Medical Sciences, Babol, Iran; ^4^ Department of Gynecology and Obstetrics, School of Medicine, Babol University of Medical Sciences, Babol, Iran; ^5^ Department of Pathology, School of Medicine, Babol University of Medical Sciences, Babol, Iran; ^6^ Infertility and Reproductive Health Research Center, Health Research Institute, Babol University of Medical Sciences, Babol, Iran; ^7^ Cellular and Molecular Biology Research Center, Health Research Institute, Babol University of Medical Sciences, Babol, Iran

**Keywords:** cervical cancer, human papillomavirus, Epstein-Barr virus, co-infection, cervical lesions

## Abstract

**Introduction:**

High-risk human papillomaviruses (HR-HPVs) are known to contribute to cervical cancer (CC), but the role of Epstein-Barr virus (EBV) in this process remains unclear, despite EBV’s widespread detection in premalignant and malignant cervical tissues.

**Methods:**

In this cross-sectional study of 258 cervical samples, including both formalin-fixed paraffin-embedded (FFPE) and fresh cervical tissues, the presence and viral load of HR-HPVs (HPV-16 and HPV-18) and EBV were evaluated in Iranian women with cervical intraepithelial neoplasia (CIN), squamous cell carcinoma (SCC), and a cervicitis control group using real-time PCR.

**Results:**

The study revealed a significant correlation between disease severity and both increased HPV-16 positivity and HPV-16 and HPV-18 co-infection (p<0.001). Interestingly, the control group had a higher frequency of EBV-positive cases than SCC/CIN groups (p<0.001). HPV-16 DNA load increased with disease severity (P<0.001), while HPV-18 showed no significant difference (P=0.058). The control group had a higher EBV DNA load compared to SCC/CIN groups (P=0.033). HPV-16 increased the risk of CIN II, CIN III, and SCC, while HPV-18 increased the risk of CIN II and CIN III. Notably, EBV was associated with a lower risk of CIN groups and SCC.

**Conclusions:**

No significant difference in EBV co-infection with HPV-16/18 was found, failing to support the hypothesis that EBV is a cofactor in CC. However, high EBV viral load in the control group suggests a potential “hit and run hypothesis” role in CC progression. This hypothesis suggests that EBV may contribute briefly to the initiation of CC with an initial impact but then becomes less actively involved in its ongoing progression.

## 1 Introduction

Cervical cancer (CC) ranks as the third most frequently diagnosed cancer and the fourth leading cause of cancer-related deaths in women globally (1). According to the 2020 CC report by the World Health Organization (WHO), there were approximately 604,127 diagnosed cases and 341,831 deaths worldwide. Among these, Iran accounted for 1,056 diagnosed cases and 644 deaths (1). The strong link between high-risk human papillomaviruses (HR-HPVs) and CC is widely acknowledged (2). High-risk oncogenic HPV types, specifically HPV 16 and HPV 18, account for a large majority (99.7%) of both low-grade cervical intraepithelial neoplasia (CIN-I) and high-grade (CIN-II/III) cervical lesions, highlighting their substantial involvement in the development of CC (3–7). Prospective population-based studies confirmed that HPV-16 is the most oncogenic type worldwide. In addition, other carcinogenic HPV types, such as HPV-18, 31, 33, 45, 52, and 58 appear to be associated with CC and other cancers in both gender (8, 9). Hence, the licensed nonavalent HPV vaccine should be used in individuals aged 9 to 45 years to prevent HPV associated cancers. In Iran, the standard CC screening system is based on American College of Obstetricians and Gynecologists (ACOG) guideline. This guideline recommends no CC screening for women younger than 21 years of age, cytology-based Pap smear screening every 3 years for women 21 to 29 years of age and co-testing with cytology and an HPV molecular detection assay every 5 years for women 30 to 65. Screening is no longer recommended in post-hysterectomy patients and after 65 years of age if the woman has a history of adequate screening (10). In addition, colposcopic follow-up is recommended for women with low-grade squamous intraepithelial lesion (LSIL), high-grade squamous intraepithelial lesion (HSIL) or atypical squamous cells of undetermined significance (ASCUS) and a positive HPV test. Moreover, in women with normal Pap smear but positive HR-HPV test colposcopy should be considered (11).

Since 1976, it has been widely acknowledged that HPV infections in the cervix are commonly linked to intraepithelial neoplasia and the development of various histological variants of invasive squamous cell carcinomas (SCC), including large-cell keratinizing, large-cell non-keratinizing, and small-cell carcinoma (12). The overexpression of HR-HPV E6 and E7 oncoproteins in cervical epithelial cells plays a pivotal role in the development of cancer (13). These oncoproteins specifically interact with tumor suppressor proteins p53 and pRB, leading to the evasion of apoptosis and disruption of the cell cycle, respectively (14, 15). The persistent expression of these viral oncoproteins in HPV-infected cells leads to the accumulation of genetic alterations and the transformation of normal cervical epithelial cells into malignant cell (16). Understanding their function has informed the development of vaccines and screening methods for CC prevention (17, 18). However, it should be noted that HR-HPV infection alone is not sufficient to cause CC, as LSIL often regress to normal or ASCUS (19, 20). Out of all LSIL cases, only 3.6% progress to HSIL (21). Interestingly, approximately half of the LSIL cases associated with HPV16 or HPV18 exhibit regression (20). Therefore, the progression of cervical lesions requires the involvement of other factors, including virus specific (e.g., integration) (22, 23) host-related and environmental elements (e.g., genetic and epigenetic alteration of cellular genes, smoking, oral contraceptives, early age of pregnancy, and multiparity) (22, 24–26). In this regard, the role of additional viral infections in the progression of CC remains unclear (27, 28). Studies have reported a synergistic effect of carcinogenic factors, where in multiple viruses interact at various stages of tumor development (12, 29).

Epstein-Barr virus (EBV), a common human gamma herpesvirus that is well-known for causing mononucleosis (12), is capable of inducing cellular transformation in cells expressing the EBV/C3d receptor (30). This receptor is present in both ecto-cervical and endo-cervical biopsies of the cervix, making the cells more susceptible to other carcinogenic factors. These discoveries indicate that EBV could potentially act as a “helper” in the progression of CC, playing a role in its advancement (30, 31). It can be transmitted sexually (32) and has the ability to replicate in cervical cells (33). Additionally, chronic cervicitis may contribute to the facilitation of EBV infection (34). EBV infection is widespread in the population (35) and has been associated with various lymphocytic and epithelial cancers, including Burkitt’s lymphoma, Hodgkin’s lymphoma, T cell lymphoma, nasopharyngeal carcinoma (NPC), and gastric adenocarcinoma (12). In addition, EBV has been implicated in extranodal NK/T cell lymphoma, nasal type, and diffuse large B cell lymphoma (36).

One of the key factors involved in EBV’s association with CC is the expression of Epstein-Barr virus-encoded small RNA (EBER), a non-coding RNA molecule produced by the virus. In CC, EBERs have been found to be present in both tumor cells and adjacent non-tumor cells. Their presence has been associated with more advanced stages of CC and poorer prognosis (29, 37).

The co-infection of HR-HPV and EBV in cervical tissues has been observed in numerous studies (12, 29, 38), while early reports have been inconsistent in reporting this association (39, 40). It is important to ascertain whether EBV plays a causal role in the development of CC or if it is merely an incidental presence, as this has significant clinical implications (41).

Previous studies have shown that HPV-16 and HPV-18 viral load values are valuable indicators for predicting CC progression (42, 43). However, research on EBV viral load in cervical samples and its association with CC is limited (3). Most previous studies on EBV used qualitative methods and did not quantify viral copy number per cell. Therefore, this study is the first to examine the co-infection of HPV-16, HPV-18, and EBV in cervical lesions among Iranian women and evaluate positive samples in terms of viral copy number per cell.

## 2 Materials and methods

### 2.1 Study population and specimen collection

The present cross-sectional study analyzed a total of 258 cervical samples, including 204 formalin‐fixed paraffin‐embedded (FFPE) and 54 fresh cervical tissues. The FFPE samples DNA were obtained from the Genome Bank of Department of Microbiology and Biotechnology affiliated to Babol University of Medical Sciences, which was linked to a previous research project identified by code 9909538 All FFPE samples were fixed in 10% neutral buffered formalin that minimizes DNA fragmentation and decreases false negative results. Furthermore, fresh tissues were collected from patients who attended the colposcopy clinic of Ayatollah Rouhani Hospital in Babol, Iran, between 2019 and 2021 by consecutive sampling. Indication criteria for cervical biopsy was according to ACOG guidelines (10). The Fresh samples were promptly submerged in tubes containing RNAlater solution and stored at -20 °C until analysis. The classification of various histological subtypes of the cervix, including CIN I, II, III, and SCC, was performed by the pathologist using the classification system for tumors of the female genital tract provided by the WHO (44). Patient medical records and anthropometric data were obtained for this study. The research received approval from the local ethics committee at Babol University of Medical Sciences in Babol, Iran, under the ethical number IR.MUBABOL.REC.1399.071. Written informed consent was obtained from all patients involved in the study.

### 2.2 DNA extraction

The current study utilized FFPE DNA samples obtained from the Genome Bank of Department of Microbiology and Biotechnology affiliated to Babol University of Medical Sciences. Additionally, DNA was extracted from fresh tissue samples using the FavorPrep ™ tissue genomic DNA extraction mini kit (Yekta Tajhiz Azma Inc., Tehran, Iran) following the instructions provided by the manufacturer. DNA extracted from the CaSki, HeLa, and B95-8 cell lines was employed as positive controls for HPV-16, HPV-18, and EBV, respectively. The extracted DNA was evaluated for both its quantity and quality using a NanoDrop spectrophotometer (Thermo Scientific, Wilmington, USA) at the end of the extraction process.

### 2.3 Detection and quantitation of HR-HPV and EBV DNA using real-time PCR assays

The real-time PCR method was used to detect and measure the amount of HPV-16 *E6*, HPV-18 *E7*, and EBV *EBER* DNA. The viral load of target genes was assessed by dividing the viral DNA copy number by half of the copy number of the *RNase P* gene, considering that each diploid cell contains two copies of the *RNase P* gene. Recombinant plasmids, containing the target gene sequences of HPV-16 *E6*, HPV-18 *E7*, and EBV *EBER*, along with the conserved region of the *RNase-P* cellular gene (Internal control), were prepared as real-time PCR standards based on previous studies (45, 46). According to a previously established protocol, quantitative real-time PCR was performed using a Rotor-Gene^®^ Q real-time PCR system (QIAGEN GmbH, Hilden, Germany). The PCR was conducted with specific primer sets and a TaqMan probe designed for target genes, as well as the human *RNase P* gene (47). Each reaction was prepared with a total volume of 25 μl, consisting of 200 ng of purified DNA, 12.5 µl of Add-Probe Taq Master 2x (Addbio, South Korea), 0.3 mM of each primer, and 0.2 mM of TaqMan probe (Metabion, Bayern, Germany). The real-time PCR cycling conditions for HPV-16 *E6*, EBV *EBER*, and *RNase P* were as follows: initial denaturation at 95°C for 3 min, followed by 40 cycles of denaturation at 95°C for 15 s and annealing/extension at 60°C for 30 s (for HPV-16 *E6* and *RNase P*), and 20 s for EBV *EBER*. For HPV-18 *E7*, the thermal cycles included initial denaturation at 95°C for 3 min, followed by 40 cycles of denaturation at 95°C for 10 s, annealing at 54.5°C for 45 s, and extension at 72°C for 45 s. To generate standard curves, real-time PCR was performed on a 10-fold dilution series of recombinant purified plasmids containing the target genes and the internal control gene. The dilution series ranged from 13 × 10^2^ to 13 × 10^6^ copies/μl.

### 2.4 Statistical analysis

The statistical analysis was conducted using SPSS Version 23 (SPSS Inc., Chicago, IL, USA). Differences in proportions between categorical variables were analyzed using the chi-square or Fisher’s exact test. The non-parametric statistical test of Kruskal-Wallis was applied to analyze continuous variables. Additionally, the odds ratio (OR) and the corresponding 95% Confidence Interval (CI) were estimated using a Bayesian logistic regression model. *P* values less than 0.05 were considered statistically significant in all analyses.

## 3 Results

The current cross-sectional study involved the analysis of 258 cervical samples, including 204 FFPE tissues and 54 fresh cervical tissues. Histopathological examination resulted in the classification of the 54 fresh cervical tissues into distinct groups, which included a control group comprising 39 (72.2%) cases of cervicitis, 4 (7.4%) cases of CIN I, 6 (11.1%) cases of CIN II, 4 (7.4%) cases of CIN III, and 1 (1.9%) case of SCC. Similarly, the 204 FFPE cervical tissues were categorized into specific groups, consisting of a control group with 102 (50%) cases of cervicitis, 22 (10.8%) cases of CIN I, 23 (11.3%) cases of CIN II, 32 (15.7%) cases of CIN III, and 25 (12.3%) cases of SCC. The patients’ mean age was 40.01 ± 11.19 years, ranging from 16 to 90 years. The patients’ mean age ± standard deviation (SD) and age range in different types of cervical injuries were as follows: patients with chronic cervicitis (44.16 ± 12.77) (age range:21 to 77 years), cases of CIN I (36.59 ± 10.31) (age range:21 to 61 years), CIN II (31.96 ± 6.36) (age range:16 to 42 years), CIN III (40.69 ± 14.27) (age range:23 to 90 years) and SCC (49.28 ± 14.18) (age range:25 to 75 years). There was a significant difference in the mean age among all the pathological groups (p<0.001). Additionally, pairwise comparison of the groups, using Bonferroni’s *post hoc* test, revealed significant differences in patients’ ages between the CIN II and control group (p-value < 0.001), CIN I and SCC group (p-value = 0.009), and CIN II and SCC groups (p-value < 0.001).

In the current study, the frequencies of HPV-16, HPV-18, and EBV-positive cases in different pathological groups were examined using real-time PCR tests, and the results are summarized in **Table 1**. The results demonstrated a significant correlation between disease severity and an increased prevalence of HPV-16 positivity (p<0.001). However, the percentages of HPV-18 positive cases did not differ significantly among the groups (p=0.065). Conversely, the control group exhibited a significantly higher prevalence of EBV-positive cases compared to the other groups, indicating the highest incidence of EBV in chronic cervicitis (p<0.001). On the other hand, no significant differences were observed in the rates of co-infection between EBV and HPV-16 (p=0.212) or EBV and HPV-18 (p=0.616) among the groups, and no triple infections of HPV-16, HPV-18, and EBV were detected in the cervical samples. Notably, a significant association was found between HPV-16 and HPV-18 co-infection patterns (p<0.001), indicating an increase in prevalence with disease severity.

**Table 1:** Prevalence of HR-HPV types and EBV in different histopathological groups of cervical disease.

Histopathological Groups						
Virus	SCC	CIN III	CIN II	CIN I	Chronic Cervicitis	p-value
**HPV-16 Positive**	19 (73.07%)	25 (69.45%)	15 (51.72%)	9 (34.61%)	28 (19.85%)	<0.001^*^
**HPV-16 Negative**	7 (26.93%)	11 (30.55%)	14 (48.28%)	17 (65.39%)	113 (19.85%)	
**HPV-18 Positive**	6 (23.07%)	11 (30.55%)	10 (34.48%)	7 (26.92%)	21 (14.89%)	0.065
**HPV-18 Negative**	20 (76.93%)	25 (69.45%)	19 (65.52%)	19 (73.08%)	120 (85.11%)	
**EBV Positive**	5 (19.23%)	3 (8.34%)	0 (0%)	2 (7.96%)	68 (48.22%)	<0.001^*^
**EBV Negative**	21 (80.77%)	33 (91.66%)	29 (100%)	24 (92.31%)	73 (51.78%)	
**HPV-16 and** **HPV-18** **Positive**	3 (11.53%)	8 (22.23%)	4 (13.79%)	1 (3.84%)	4 (2.83%)	0.001^*^
**HPV-16 and** **HPV-18** **Negative**	23 (88.47%)	28 (77.77%)	25 (86.21%)	25 (96.16%)	137 (97.17%)	
**HPV-16 and EBV** **Positive**	3 (11.53%)	0 (0%)	0 (0%)	0 (0%)	18 (12.76%)	0.212
**HPV-16 and EBV Negative**	23 (88.47%)	36 (100%)	29 (100%)	26 (100%)	123 (87.24%)	
**HPV-18 and EBV Positive**	2 (7.69%)	3 (8.34%)	0 (0%)	1 (3.84%)	8 (5.67%)	0.616
**HPV-18 and EBV Negative**	24 (92.31%)	33 (91.66%)	29 (100%)	2529 (96.16%)	133 (94.33%)	

* means statistically significant.

**Figure 1:**
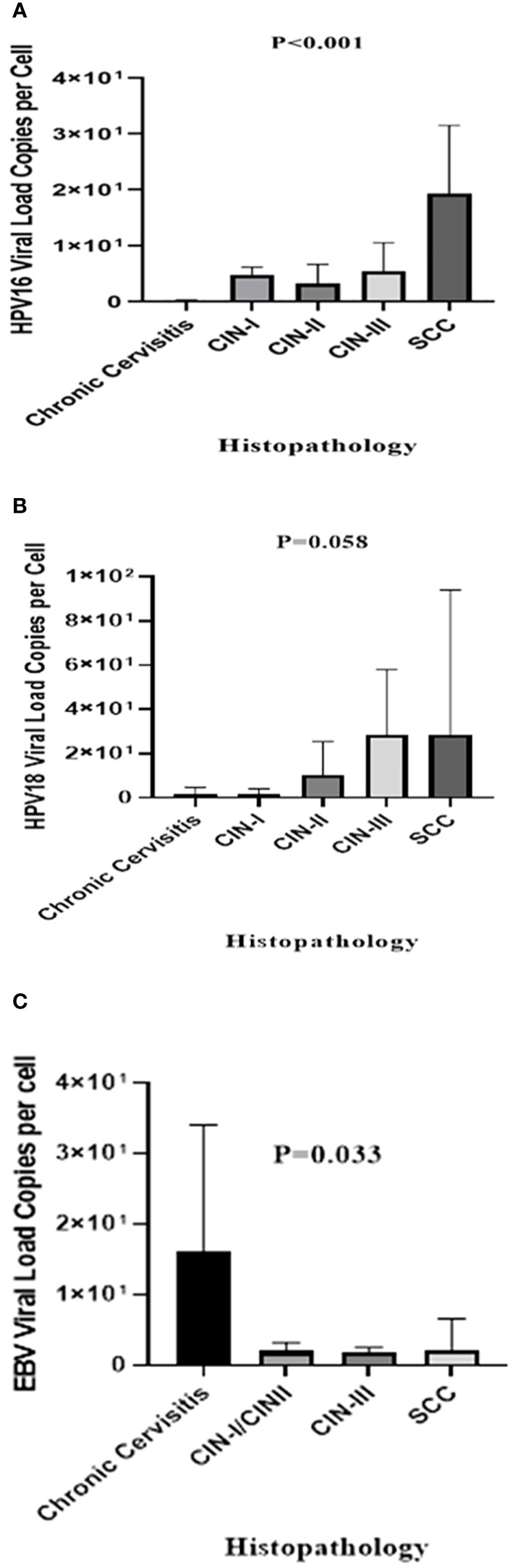
The mean viral loads of HPV-16 **(A)**, HPV-18 **(B)**, and EBV **(C)** in different histopathological groups (copies/cell). The *P*-value was determined by the Kruskal-Wallis test.

Using a known single-copy gene, human *RNase P*, the DNA load of HPV-16, HPV-18, and EBV was normalized as the viral copy number per cell. In HPV-16 positive samples, control group exhibited a significantly lower mean HPV-16 copy number (0.21 ± 0.53 copies/cell) compared to CIN I (4.78 ± 4.13 copies/cell, P = 0.001), CIN II (3.24 ± 3.4 copies/cell, P = 0.003), CIN III (10.01 ± 13.54 copies/cell, P = 0.001), and the SCC samples (19.28 ± 25.3 copies/cell, P < 0.001) (**Figure 1A**). Regarding HPV-18, the mean copy numbers in SCC, CIN I, CIN II, CIN III, and the control group were 28.76 ± 65.4, 1.88 ± 2.36, 10.16 ± 15.42, 28.71 ± 29.31, and 1.9 ± 2.78 copies/cell, respectively. No statistically significant difference was observed in HPV-18 copy numbers among the groups (P = 0.058) (**Figure 1B**), except for a significant difference between the control group and the CIN-III (P = 0.013) and SCC groups (P = 0.013). On the other hand, EBV positive samples exhibited a higher mean copy number in the control group (16.05 ± 17.96 copies/cell) compared to SCC (2.09 ± 4.47 copies/cell), CIN I/II (1.93 ± 1.29 copies/cell), and CIN III (1.92 ± 0.55 copies/cell) samples, indicating a statistically significant difference (P = 0.033) (**Figure 1C**).

In this study, Bayesian logistic regression was used to estimate the association between HPV-16, HPV-18, and EBV infections and the risk of CC development at different stages. The odds ratios (OR) for each infection are presented in **Table 2**. The results revealed that HPV-16 infection significantly increased the likelihood of developing CIN II (OR=5.54), CIN III (OR=10.21), and SCC (OR=10.62) compared to the control group. HPV-18 infection was also associated with a higher risk of CIN II (OR=4.51) and CIN III (OR=3.55). Interestingly, EBV infection showed a statistically significant reduction in the risk of developing CIN I, CIN II, CIN III, and SCC (p ≤ 0.001).

**Table 2:** The estimated odds ratios of HPV-16, HPV-18 and EBV infections in cervical disease.

	Histopathological Groups							
	CIN I		CIN II		CIN III		SCC	
	OR^a^ (95% CI^b^)	P value	OR (95% CI)	P value	OR (95% CI)	P value	OR (95% CI)	P value
**Age**	0.94 (0.89-0.98)	0.006	0.88 (0.83-0.93)	0.001>	0.97 (0.93-1.01)	0.145	1.03 (0.99-1.07)	0.127
**HPV-16**	2.36 (0.86-6.47)	0.096	5.54 (1.98-15.47)	0.001	10.21 (4-26.01)	<0.001	10.62 (3.72-30.31)	0.001>
**HPV-18**	2.41 (0.82-7.12)	0.111	4.51 (1.49-13.62)	0.008	3.55 (1.30-9.70)	0.013	2.47 (0.77-7.94)	0.128
**EBV**	0.05 (0.01-0.23)	<0.001^*^	0.07 (0.02-0.18)	0.001^*^>	0.05 (0.01-0.20)	0.001^*^>	0.12 (0.04-0.40)	0.001^*^

CI, confidence interval; OR, odds ratio.

* means statistically significant.

^a^ means odds ratio; ^b^ means confidence interval.

## 4 Discussion

The long interval between HR-HPV infection and CC development suggests the involvement of other etiologic agents in malignancy progression. In light of this, our study aimed to explore the potential role of EBV in conjunction with HR-HPV types 16 and 18 co-infections among Iranian women with cervical disease. Understanding the interplay of these viral co-infections could provide crucial insights into the complex pathogenesis of CC in this population.

This study indicated a strong correlation between disease severity and both increased HPV-16 positivity and HPV-16 and HPV-18 co-infection. These results are consistent with previous studies showing that HPV-16 is the most prevalent HR-HPV type associated with CC. For instance, investigations by Smith et al. (48) in Australia and a meta-analysis by Zhang et al. (49) involving multiple studies worldwide consistently reported a significant association between HPV-16 infection and CC development. These findings collectively reinforce the notion that HPV-16 plays a prominent role in the pathogenesis of CC across diverse populations.

Unlike HPV-16, the prevalence of HPV-18 did not vary significantly among different pathological groups in this study, similar to previous research conducted in Iran (50). HPV-18, another HR-HPV type, exhibited a relatively consistent prevalence across CIN and SCC cases, suggesting that its contribution to disease severity may be less pronounced compared to HPV-16. However, it should be noted that the sample size for HPV-18 positive cases was relatively small, which may have affected the statistical power to detect significant differences. Studies have reported conflicting findings on the association between HPV-18 and cervical disease (51–53), indicating population variability and the need for further investigation.

In contrast to HR-HPVs, EBV exhibited a lower prevalence in pre-malignant/malignant samples. The control group showed a higher incidence of EBV-positive cases (48.22%) compared to the SCC (19.23%) and CIN groups (ranging from 0-8.34%). This finding suggests a potential protective effect of EBV against the development of cervical disease. In contrast, other studies have reported findings supporting a potential association between EBV and CC. For instance, a study by Joharinia et al. (54) conducted in the Southwest of Iran reported an elevated frequency of EBV infection in HISL than LSIL and suggested EBV as an enhancing factor in disease progression. Similarly, studies conducted in other populations, such as studies by Landers et al. (55) and Sasagawa et al. (37), have reported a higher prevalence of EBV in CC cases compared to controls, supporting a potential role of EBV in disease development. The discrepancy between this study and previous studies may be attributed to variations in study designs, sample sizes, and detection methods.

Also, this study did not find a significant association between HPV-16/18 and EBV co-infection, indicating that EBV may not act as a cofactor in CC development. Further follow-up of patients with chronic cervicitis, specifically regarding the co-infection of EBV and HPV16/18, is crucial to gain more precise insights. Studies indicate that EBV infection raises the risk of HPV-16 genome integration, potentially contributing to CC development (38, 56). Moreover, in samples positive for both EBV and HPV, there is an increased methylation of the RB1 and E-cadherin (CDH1) gene promoters compared to tumors that are negative for EBV but positive for HPV. Methylation of these genes may play a role in the development of CC (57). Differences in EBV and HPV genotypes across populations could also contribute to variable results. More research is needed to clarify the roles of EBV and specific HPV genotypes in CC development. However, no triple infections of HPV-16, HPV-18, and EBV were detected, limiting insights into their interaction. Larger studies are necessary to understand the relationship between these viruses in cervical disease.

Also, logistic regression analysis confirmed the prevalence findings, with HPV-16 infection significantly increasing the risk of CIN II, CIN III, and SCC, and HPV-18 infection associated with a higher risk of CIN II and CIN III. Conversely, EBV infection was found to significantly reduce the risk of CIN groups and SCC. These results suggest that HPV-16 plays a prominent role in disease progression, while EBV may have a protective effect against the development of CC.

In this study, Quantitative Real-Time PCR was utilized to quantify the copy numbers per cell of HPV-16, HPV-18, and EBV in non-cancerous control group, pre-malignant, and malignant cervical samples. Previous research has suggested that the viral load values of HPV-16 and HPV-18 can serve as valuable indicators for predicting CC progression (42, 58, 59). The study identified a significant difference in the mean HPV-16 viral load per cell among the groups. Disease severity consistently exhibited a positive correlation with an increased mean HPV-16 viral load per cell, consistent with previous studies (60, 61). Additionally, a statistically significant difference was found in the mean HPV-18 viral load per cell between the control group and the CIN-III and SCC groups, providing further support to findings reported by other research groups globally (62, 63). Limited research has been conducted on the EBV viral load in cervical samples, and its potential association with CC remains poorly understood (3). Surprisingly, the study revealed a significantly higher EBV viral load per cell in the control group compared to the pre-malignant and malignant groups. Higher EBV copy number in cervicitis group compared to CIN/SCC suggests a potential “hit and run hypothesis” role in cervical carcinogenesis. The hit and run phenomenon suggest that EBV may initiates the process of CC formation (the hit) but as mutations are accumulated over time then becomes less actively involved in its ongoing progression (the run). It is noteworthy that in the current study, the absence of a clear dose-response pattern between EBV copy number in pre-malignant (CINs) and malignant (SCC) groups and the cross-sectional design with imbalance in patients numbers in the groups makes it difficult to interpret the role of hit and run phenomenon in cervical carcinogenesis. However, larger epidemiologic studies that investigate EBV viral load in cervical samples are necessary to draw a definitive conclusion.

The findings of the current investigation should be interpreted with caution due to some limitations, including the small sample sizes, cross-sectional design, the lack of a normal cervical sample as a control group, and the potential for false-positive identification of the EBV genome due to infiltration of EBV-infected lymphocytes into the cervical tissue. On the other hand, conducting additional complementary studies, including assessing oncogene expression at the RNA and protein levels, investigating the interaction between oncogenes and tumor suppressor proteins using immunohistochemistry (IHC), and performing whole genome sequencing of cancerous and non-cancerous cells to evaluate possible mutations, can provide further insights into the potential tumorigenic role of these viruses in the development of CC.

## 5 Conclusion

This study provides valuable insights into the prevalence, viral load, and risk of CC associated with HR-HPVs and EBV in Iranian women with cervical disease. The high prevalence of HPV-16 and HPV-18 reaffirms their significant role in disease development, with HPV-16 showing a stronger association with disease severity. In contrast, EBV exhibits a lower prevalence and potential protective effect in this population, suggesting a more complex relationship. Taken together, the interpretation of our findings in terms of role of EBV and HR-HPVs Co-infections in cervical carcinogenesis should be interpreted with caution due to cross-sectional design of current study and further investigations with case-control or cohort design is necessary.

## Data availability statement

The original contributions presented in the study are included in the article/supplementary material. Further inquiries can be directed to the corresponding authors.

## Ethics statement

The studies involving humans were approved by Babol University of Medical Sciences. The studies were conducted in accordance with the local legislation and institutional requirements. The participants provided their written informed consent to participate in this study.

## Author contributions

FarzaneS: Investigation, Writing – original draft. TM: Investigation, Writing – original draft. MT: Investigation, Writing – review & editing. SY: Writing – review & editing. MJ: Writing – review & editing. MR: Writing – review & editing. HG: Writing – review & editing. ZB: Writing – review & editing, Supervision, Project administration, Methodology, Conceptualization. FarzinS: Writing – review & editing, Supervision, Project administration, Methodology, Conceptualization.

## References

[1] Sung H Ferlay J Siegel RL Laversanne M Soerjomataram I Jemal A. Global cancer statistics 2020: GLOBOCAN estimates of incidence and mortality worldwide for 36 cancers in 185 countries. CA Cancer J Clin. (2021) 71:209–49. doi: 10.3322/caac.21660 33538338

[2] Anderson L O’Rorke M Jamison J Wilson R Gavin A. Prevalence of human papillomavirus in women attending cervical screening in the UK and Ireland: new data from northern Ireland and a systematic review and meta-analysis. J Med Virol. (2013) 85:295–308. doi: 10.1002/jmv.23459 23161367

[3] Blanco R Carrillo-Beltrán D Osorio JC Calaf GM Aguayo F. Role of Epstein-Barr virus and human papillomavirus coinfection in cervical cancer: epidemiology, mechanisms and perspectives. Pathogens. (2020) 9:685. doi: 10.3390/pathogens9090685 32839399 PMC7557835

[4] Izaaks CD Truter EJ Khan S. Prevalence of human papilloma virus in cytological abnormalities: Association of risk factors and cytomorphological findings. Cytojournal. (2012) 9:19. doi: 10.4103/1742-6413.100123 22993533 PMC3440928

[5] Ripabelli G Grasso GM Del Riccio I Tamburro M Sammarco ML. Prevalence and genotype identification of human papillomavirus in women undergoing voluntary cervical cancer screening in Molise, Central Italy. Cancer Epidemiol. (2010) 34:162–67. doi: 10.1016/j.canep.2009.12.010 20080070

[6] Steele JC Mann CH Rookes S Rollason T Murphy D Freeth MG. T-cell responses to human papillomavirus type 16 among women with different grades of cervical neoplasia. Br J Cancer. (2005) 93:248–59. doi: 10.1038/sj.bjc.6602679 PMC2361543 15986031

[7] Van Doorslaer K Chen Z Bernard H-U Chan PK DeSalle R Dillner J. ICTV virus taxonomy profile: Papillomaviridae. J Gen Virol. (2018) 99:989–90. doi: 10.1099/jgv.0.001105 PMC6171710 29927370

[8] Sammarco ML Ucciferri C Tamburro M Falasca K Ripabelli G Vecchiet J. High prevalence of human papillomavirus type 58 in HIV infected men who have sex with men: A preliminary report in Central Italy. J Med Virol. (2016) 88:911–4. doi: 10.1002/jmv.24406 26467111

[9] Sand FL Munk C Frederiksen K Junge J Iftner T Dehlendorff C. Risk of CIN3 or worse with persistence of 13 individual oncogenic HPV types. Int J Cancer. (2019) 144:1975–82. doi: 10.1002/ijc.31883 30246864

[10] Volerman A Cifu AS. Cervical cancer screening. Jama. (2014) 312:2279–80. doi: 10.1001/jama.2014.14992 25461998

[11] Perkins RB Adcock R Benard V Cuzick J Waxman A Howe J. Clinical follow-up practices after cervical cancer screening by co-testing: A population-based study of adherence to U.S. guideline recommendations. Prev Med. (2021) 153:106770. doi: 10.1016/j.ypmed.2021.106770 34416221 PMC8595756

[12] Khenchouche A Sadouki N Boudriche A Houali K Graba A Ooka T. Human Papillomavirus and Epstein-Barr virus co-infection in Cervical Carcinoma in Algerian women. Virol J. (2013) 10:340. doi: 10.1186/1743-422X-10-340 24252325 PMC4225508

[13] Vats A Trejo-Cerro O Thomas M Banks L. Human papillomavirus E6 and E7: What remains? Tumour Virus Res. (2021) 11:200213. doi: 10.1016/j.tvr.2021.200213 33716206 PMC7972986

[14] Boyer SN Wazer DE Band V. E7 protein of human papilloma virus-16 induces degradation of retinoblastoma protein through the ubiquitin-proteasome pathway. Cancer Res. (1996) 56:4620–4. 8840974

[15] Scheffner M Werness BA Huibregtse JM Levine AJ Howley PM. The E6 oncoprotein encoded by human papillomavirus types 16 and 18 promotes the degradation of p53. Cell. (1990) 63:1129–36. doi: 10.1016/0092-8674(90)90409-8 2175676

[16] Roberts S Young LS. Role of HPV in cervical carcinogenesis. Vaccines for the prevention of cervical cancer. USA: Oxford University Press (2008). doi: 10.1093/med/9780199543458.001.0001

[17] Akhatova A Chan CK Azizan A Aimagambetova G. The efficacy of therapeutic DNA vaccines expressing the human papillomavirus E6 and E7 oncoproteins for treatment of cervical cancer: systematic review. Vaccines. (2022) 10:53. doi: 10.3390/vaccines10010053 PMC8780177 35062714

[18] Zhang S-K Guo Z Wang P Kang L-N Jia M-M Wu Z-N. The potential benefits of HPV E6/E7 mRNA test in cervical cancer screening in China. Front Oncol. (2020) 10:533253. doi: 10.3389/fonc.2020.533253 33123463 PMC7567165

[19] Blanco R Carrillo-Beltrán D Muñoz JP Osorio JC Tapia JC Burzio VA. Characterization of high-risk HPV/EBV co-presence in pre-malignant cervical lesions and squamous cell carcinomas. Microorganisms. (2022) 10. doi: 10.3390/microorganisms10050888 PMC9144326 35630333

[20] Silveira FA Almeida G Furtado YL Cavalcanti S Silva KS Maldonado P. The association of HPV genotype with the regression, persistence or progression of low-grade squamous intraepithelial lesions. Exp Mol Pathol. (2015) 99:702–06. doi: 10.1016/j.yexmp.2015.11.001 26546836

[21] Schlecht NF Platt RW Duarte-Franco E Costa MC Sobrinho JP Prado JC. Human papillomavirus infection and time to progression and regression of cervical intraepithelial neoplasia. J Natl Cancer Inst. (2003) 95:1336–43. doi: 10.1093/jnci/djg037 12953088

[22] D. Burk R Chen Z Saller C Tarvin K L. Carvalho A Scapulatempo-Neto C. Integrated genomic and molecular characterization of cervical cancer. Nature. (2017) 543:378–84. doi: 10.1038/nature21386 PMC5354998 28112728

[23] Klaes R Woerner SM Ridder R Wentzensen N Duerst M Schneider A. Detection of high-risk cervical intraepithelial neoplasia and cervical cancer by amplification of transcripts derived from integrated papillomavirus oncogenes. Cancer Res. (1999) 59:6132–6. 10626803

[24] Appleby P Beral V Berrington de González A Colin D Franceschi S Goodhill A. Cervical cancer and hormonal contraceptives: collaborative reanalysis of individual data for 16,573 women with cervical cancer and 35,509 women without cervical cancer from 24 epidemiological studies. Lancet. (2007) 370:1609–21. doi: 10.1016/S0140-6736(07)61684-5 17993361

[25] Castellsagué X Muñoz N. Chapter 3: Cofactors in human papillomavirus carcinogenesis–role of parity, oral contraceptives, and tobacco smoking. J Natl Cancer Inst Monogr. (2003) 31):20–8. doi: 10.1093/oxfordjournals.jncimonographs.a003477 12807941

[26] Verlaat W Van Leeuwen RW Novianti PW Schuuring E Meijer C van der Zee AGJ. Host-cell DNA methylation patterns during high-risk HPV-induced carcinogenesis reveal a heterogeneous nature of cervical pre-cancer. Epigenetics. (2018) 13:769–78. doi: 10.1080/15592294.2018.1507197 PMC6224221 30079796

[27] Al-Thawadi H Ghabreau L Aboulkassim T Yasmeen A Vranic S Batist G. co-incidence of epstein–Barr Virus and high-risk human Papillomaviruses in cervical cancer of Syrian Women. Front Oncol. (2018) 8:250. doi: 10.3389/fonc.2018.00250 30035100 PMC6043788

[28] Imajoh M Hashida Y Nemoto Y Oguri H Maeda N Furihata M. Detection of Merkel cell polyomavirus in cervical squamous cell carcinomas and adenocarcinomas from Japanese patients. Virol J. (2012) 9:1–9. doi: 10.1186/1743-422X-9-154 22876976 PMC3545865

[29] Aromseree S Pientong C Swangphon P Chaiwongkot A Patarapadungkit N Kleebkaow P. Possible contributing role of Epstein-Barr virus (EBV) as a cofactor in human papillomavirus (HPV)-associated cervical carcinogenesis. J Clin Virol. (2015) 73:70–6. doi: 10.1016/j.jcv.2015.10.015 26551071

[30] Young LS Dawson CW Brown KW Rickinson AB. Identification of a human epithelial cell surface protein sharing an epitope with the C3d/Epstein-Barr virus receptor molecule of B lymphocytes. Int J Cancer. (1989) 43:786–94. doi: 10.1002/ijc.2910430508 2469656

[31] Se Thoe SY Wong KK Pathmanathan R Sam CK Cheng HM Prasad U. Elevated secretory IgA antibodies to Epstein-Barr virus (EBV) and presence of EBV DNA and EBV receptors in patients with cervical carcinoma. Gynecol Oncol. (1993) 50:168–72. doi: 10.1006/gyno.1993.1187 8397152

[32] Näher H Gissmann L Freese UK Petzoldt D Helfrich S. Subclinical Epstein-Barr virus infection of both the male and female genital tract–indication for sexual transmission. J Invest Dermatol. (1992) 98:791–3. 10.1111/1523-1747.ep12499958 1314867

[33] Sixbey JW Lemon SM Pagano JS. A second site for Epstein-Barr virus shedding: the uterine cervix. Lancet. (1986) 2:1122–4. doi: 10.1016/S0140-6736(86)90531-3 2877273

[34] Silver MI Paul P Sowjanya P Ramakrishna G Vedantham H Kalpana B. Shedding of Epstein-Barr virus and cytomegalovirus from the genital tract of women in a periurban community in Andhra Pradesh, India. J Clin Microbiol. (2011) 49:2435–39. doi: 10.1128/JCM.02206-10 PMC3147891 21525227

[35] Macsween KF Crawford DH. Epstein-Barr virus-recent advances. Lancet Infect Dis. (2003) 3:131–40. doi: 10.1016/S1473-3099(03)00543-7 12614729

[36] Montes-Mojarro IA Fend F Quintanilla-Martinez L. EBV and the pathogenesis of NK/T cell lymphoma. Cancers (Basel). (2021) 13. doi: 10.3390/cancers13061414 PMC8003370 33808787

[37] Sasagawa T Shimakage M Nakamura M Sakaike J Ishikawa H Inoue M. Epstein-Barr virus (EBV) genes expression in cervical intraepithelial neoplasia and invasive cervical cancer: a comparative study with human papillomavirus (HPV) infection. Hum Pathol. (2000) 31:318–26. doi: 10.1016/S0046-8177(00)80245-2 10746674

[38] Kahla S Oueslati S Achour M Kochbati L Chanoufi MB Maalej M. Correlation between ebv co-infection and HPV16 genome integrity in Tunisian cervical cancer patients. Braz J Microbiol. (2012) 43:744–53. doi: 10.1590/S1517-83822012000200039 PMC3768824 24031886

[39] Elgui de Oliveira D Furtado Monteiro TA Alencar de Melo W Amaral Rebouças Moreira M Alvarenga M Bacchi CE. Lack of Epstein-Barr virus infection in cervical carcinomas. Arch Pathol Lab Med. (1999) 123:1098–100. doi: 10.5858/1999-123-1098-LOEBVI 10539915

[40] Yang YY Koh LW Tsai JH Tsai CH Wong EF Lin SJ. Correlation of viral factors with cervical cancer in Taiwan. J Microbiol Immunol Infect. (2004) 37:282–7. 15497009

[41] Shi Y Peng SL Yang LF Chen X Tao YG Cao Y. Co-infection of Epstein-Barr virus and human papillomavirus in human tumorigenesis. Chin J Cancer. (2016) 35:16. doi: 10.1186/s40880-016-0079-1 26801987 PMC4724123

[42] Gao D Zhang Y Zhu M Liu S Wang X. miRNA expression profiles of HPV-infected patients with cervical cancer in the Uyghur population in China. PloS One. (2016) 11:e0164701. doi: 10.1371/journal.pone.0164701 27764149 PMC5072605

[43] van der Weele P van Logchem E Wolffs P van den Broek I Feltkamp M de Melker H. Correlation between viral load, multiplicity of infection, and persistence of HPV16 and HPV18 infection in a Dutch cohort of young women. J Clin Virol. (2016) 83:6–11. doi: 10.1016/j.jcv.2016.07.020 27522635

[44] Nayar R Wilbur DC. The Pap test and Bethesda 2014. Cancer Cytopathol. (2015) 123:271–81. doi: 10.1002/cncy.21521 25931431

[45] Zebardast A Yahyapour Y Majidi MS Chehrazi M Sadeghi F. Detection of Epstein-Barr virus encoded small RNA genes in oral squamous cell carcinoma and non-cancerous oral cavity samples. BMC Oral Health. (2021) 21:502. doi: 10.1186/s12903-021-01867-8 34615503 PMC8495909

[46] Setayeshi S Yahyapour Y Ghorbani H Nokhostin F Bagheri M Sadeghi F. Development of a Diagnostic Panel to Measure the Viral Load and the Physical Status of the Human Papilloma Virus16 genome using Multiplex Real-Time PCR Method. J Knowl Health Basic Med Sci. (2023) 18:9–19. doi: 10.22100/jkh.v18i2.3048

[47] Saber Amoli S Hasanzadeh A Sadeghi F Chehrazi M Seyedmajidi M Zebardast A. Prevalence of co-infection by human papillomavirus, Epstein- Barr virus and Merkel cell polyomavirus in Iranian oral cavity cancer and pre-malignant lesions. Int J Mol Cell Med. (2022) 11:64–77. doi: 10.22088/IJMCM.BUMS.11.1.64 36397808 PMC9653548

[48] Smith MA Sherrah M Sultana F Castle PE Arbyn M Gertig D. National experience in the first two years of primary human papillomavirus (HPV) cervical screening in an HPV vaccinated population in Australia: observational study. BMJ. (2022) 376. doi: 10.1136/bmj-2021-068582 PMC8965648 35354610

[49] Zhang J Cheng K Wang Z. Prevalence and distribution of human papillomavirus genotypes in cervical intraepithelial neoplasia in China: a meta-analysis. Arch Gynecol Obstet. (2020) 302:1329–37. doi: 10.1007/s00404-020-05787-w PMC7584548 32914222

[50] Joharinia N Farhadi A Hosseini SY Safaei A Sarvari J. Association of HPV16 and 18 genomic copies with histological grades of cervical lesions. Virus Dis. (2019) 30:387–93. doi: 10.1007/s13337-019-00545-2 PMC6863989 31803806

[51] de Martel C Plummer M Vignat J Franceschi S. Worldwide burden of cancer attributable to HPV by site, country and HPV type. Int J Cancer. (2017) 141:664–70. doi: 10.1002/ijc.30716 PMC5520228 28369882

[52] Ma X Yang M. The correlation between high-risk HPV infection and precancerous lesions and cervical cancer. Am J Transl Res. (2021) 13:10830–36. PMC8507010 34650762

[53] Pešut E Đukić A Lulić L Skelin J Šimić I Milutin Gašperov N. Human papillomaviruses-associated cancers: an update of current knowledge. Viruses. (2021) 13. doi: 10.3390/v13112234 PMC8623401 34835040

[54] Joharinia N Faghihinezhad S Seyedi K Farhadi A Hosseini SY Safaei A. Co-existing of HSV1/2 or EBV infection with the presence of high-risk HPV DNA in cervical lesions in the southwest of Iran. Asian Pac J Cancer. (2020) 21:1459. doi: 10.31557/APJCP.2020.21.5.1459 PMC7541875 32458656

[55] Landers RJ O’Leary JJ Crowley M Healy I Annis P Burke L. Epstein-Barr virus in normal, pre-malignant, and Malignant lesions of the uterine cervix. J Clin Pathol. (1993) 46:931–5. doi: 10.1136/jcp.46.10.931 PMC501621 8227411

[56] Ghosh S Shetty RS Pattanshetty SM Mallya SD Pandey D Kabekkodu SP. Human papilloma and other DNA virus infections of the cervix: A population based comparative study among tribal and general population in India. PloS One. (2019) 14:e0219173. doi: 10.1371/journal.pone.0219173 31247023 PMC6597196

[57] McCormick TM Canedo NH Furtado YL Silveira FA de Lima RJ Rosman AD. Association between human papillomavirus and Epstein-Barr virus DNA and gene promoter methylation of RB1 and CDH1 in the cervical lesions: a transversal study. Diagn Pathol. (2015) 10:1–7. doi: 10.1186/s13000-015-0283-3 26032781 PMC4450846

[58] Chikandiwa A Pisa PT Tamalet C Muller EE Michelow P Chersich MF. Prevalent, persistent anal HPV infection and squamous intraepithelial lesions: Findings from a cohort of men living with HIV in South Africa. PloS One. (2019) 14:e0225571. doi: 10.1371/journal.pone.0225571 31805074 PMC6894774

[59] Saunier M Monnier-Benoit S Mauny F Dalstein V Briolat J Riethmuller D. Analysis of human papillomavirus type 16 (HPV16) DNA load and physical state for identification of HPV16-infected women with high-grade lesions or cervical carcinoma. J Clin Microbiol. (2008) 46:3678–85. doi: 10.1128/JCM.01212-08 PMC2576617 18799702

[60] Álvarez-Paredes L Santibañez M Galiana A Rodríguez Díaz JC Parás-Bravo P Andrada-Becerra ME. Association of human papillomavirus genotype 16 viral variant and viral load with cervical high-grade intraepithelial lesions. Cancer Prev Res. (2019) 12:547–56. doi: 10.1158/1940-6207.CAPR-18-0397 31208965

[61] Manawapat A Stubenrauch F Russ R Munk C Krüger Kjaer S Iftner T. Physical state and viral load as predictive biomarkers for persistence and progression of HPV16-positive cervical lesions: results from a population based long-term prospective cohort study. Am J Cancer Res. (2012) 2:192–203. 22432058 PMC3304573

[62] Adcock R Cuzick J Hunt WC McDonald RM Wheeler CM Joste NE. Role of HPV genotype, multiple infections, and viral load on the risk of high-grade cervical neoplasia. Cancer Epidemiol Biomarkers Prev. (2019) 28:1816–24. doi: 10.1158/1055-9965.EPI-19-0239 PMC8394698 31488417

[63] Zhang Y Du H Xiao A Zhang W Wang C Huang X. Verification of the association of the cycle threshold (Ct) values from HPV testing on Cobas4800 with the histologic grades of cervical lesions using data from two population-based cervical cancer screening trials. Infect Agents Cancer. (2022) 17:1–12. doi: 10.1186/s13027-022-00440-4 PMC9188717 35690793

